# Retiform hemangioendothelioma: A case report and literature review

**DOI:** 10.1016/j.ijscr.2025.110869

**Published:** 2025-01-12

**Authors:** Lin Song, Dule Xing, Zhixin Cao, Yuanyuan Zong, Dongsheng Hou

**Affiliations:** Department of Pathology, Shandong Provincial Hospital Affiliated to Shandong First Medical University, Jinan, Shandong Province 250025, China

**Keywords:** Retiform hemangioendothelioma, Vascular tumor, Submandibular, Vascular endothelial, Case report

## Abstract

**Introduction and importance:**

Retiform hemangioendothelioma(RH) is a rare vascular tumor affecting patients over a wide age range without a gender predilection; only about 50 cases have been described so far.

**Case presentation:**

We report a case of submandibular retiform hemangioendothelioma in a 58-year-old woman who had been diagnosed with RH 20 years ago and had experienced recurrence four times during the past 20 years. This will increase the limited number of such cases in the hope of gaining a better understanding of this rare type of tumor. The histological features of RH are characterized by arborizing blood vessels arranged in a testicular network pattern with endothelial cells arranged in a hobnail pattern. Immunohistochemistry revealed CD31, CD34, ERG, and other vascular markers.

**Clinical discussion:**

Based on the morphological and immunohistochemical results, we diagnosed the patient with a rectiform hemangioendothelioma with local malignancy that transformed into angiosarcoma, with enlargement of the lymph nodes in the neck area, possibly indicating tumor lymph node metastasis. The patient underwent extended resection and completed 14 radiotherapy sessions. Follow-up at 3 months after surgery showed no recurrence.

**Conclusion:**

RH is a low-grade malignant intermediate vascular tumor that is prone to recurrence and does not generally metastasize to distant sites. Patients with recurrent relapses may undergo malignant transformation or lymph node metastases. Treatment primarily relies on wide excision and adjuvant radiotherapy may be necessary when required.

## Highlights

• Here, we report a rare case of retiform hemangioendothelioma(RH) in the submandibular region.
• The histological features of RH are characterized by arborizing blood vessels arranged in a testicular rete-like pattern, with endothelial cells arranged in a hobnail pattern, immunohistochemistry expresses vascular immunohistochemical markers.
• The relationship between RH and HHV-8 virus is unclear, and some RH tumors have a YAP1 rearrangement.

## 1 Introduction and importance

Retiform hemangioendothelioma(RH) is a unusual vascular tumor of intermediate grade malignancy, it was firt reported in 1994 [1], and only about 50 cases were described so far [[2], [3], [4]]. Here, we report a case of submandibular retiform hemangioendothelioma in a 58-year-old woman who had been diagnosed with RH 20 years ago and had experienced recurrence 4 times during the past 20 years. We will conduct a comprehensive analysis of the tumor's tissue morphology and immunohistochemistry. The histological features of RH are characterized by arborizing blood vessels arranged in a testicular network pattern with endothelial cells arranged in a hobnail pattern. Immunohistochemistry revealed CD31, CD34, ERG, and other vascular markers. The patient underwent an extended resection, and had completed 14 sessions of radiotherapy, currently in good condition. This paper will increase the limited number of such cases in the hope of gaining a better understanding of this rare type of tumor.

## 2 Case presentation

The patient was a 58-year-old female with a right-sided submaxillary mass that had persisted for >20 years. The patient reported discovering a peanut-sized mass in the right submandibular area 20 years ago and underwent excision at a local hospital in 1997 without pathological examination. However, due to recurrence, she had the mass removed three more times, with the third surgery taking place in May 2019, and the postoperative pathology was (facial mass) retiform hemangioendothelioma. In 2022, the mass recurred and gradually enlarged, leading the patient to seek further treatment at our hospital on September 9, 2024, and was admitted to the ward with a diagnosis of “right submandibular mass.”

**Fig. 1:**
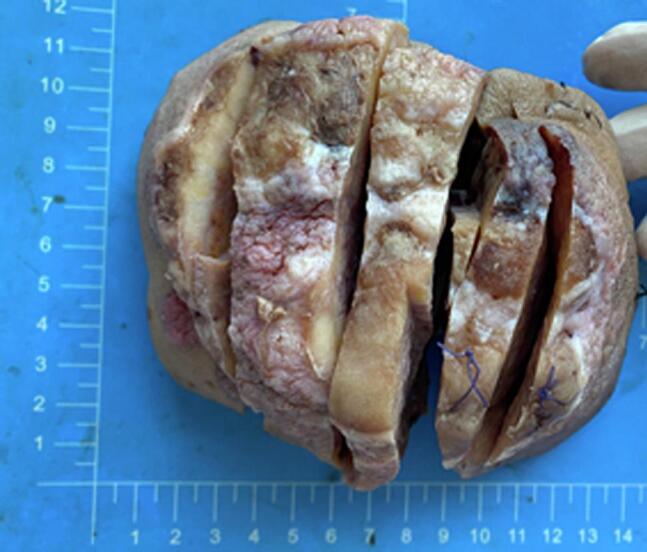
Gross image, skin surface, swelling-like growth, grayish-white, grayish-black, nodular protrusion with local surface ulceration.

**Fig. 2:**
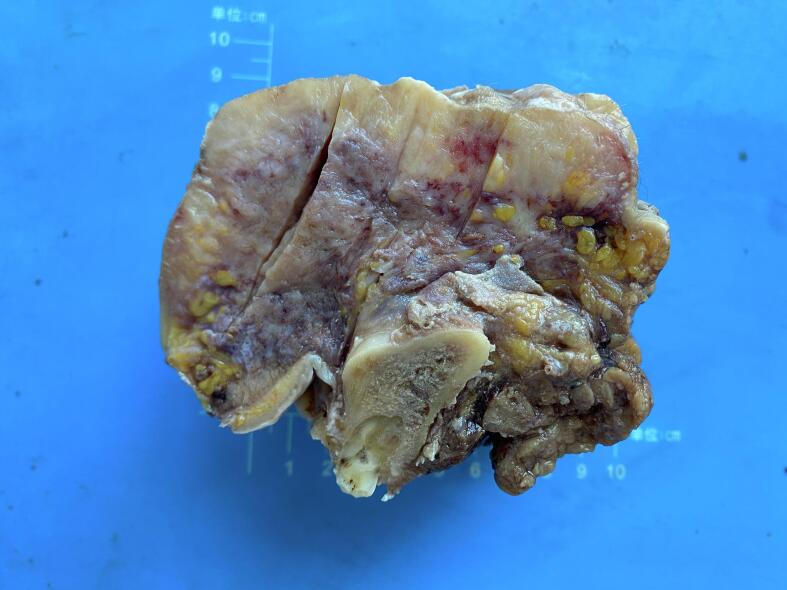
Gross image, cross-section, the tumor cut surface is grayish-white, grayish-yellow, with local bleeding, fat infiltration is visible, the tumor shows infiltrative growth, and the tumor does not invade the jawbone. (For interpretation of the references to colour in this figure legend, the reader is referred to the web version of this article.)

The patient's facial symmetry was asymmetric, with a mass measuring approximately 10 × 8 cm, visible in the right submandibular area, showing ulceration and purulent discharge. The mass was tough and mobile. No significantly enlarged lymph nodes were palpable in the bilateral submandibular or mental regions. The specimen showed a mass protruding from the epidermis, with local ulceration. The cut surface of the tumor was grayish-white and grayish-yellow, hard, and tough in texture. The tumor exhibited infiltrative growth beneath the skin, with visible fat infiltration, without involvement of the jawbone (as shown in Fig. 1, Fig. 2).

**Fig. 3:**
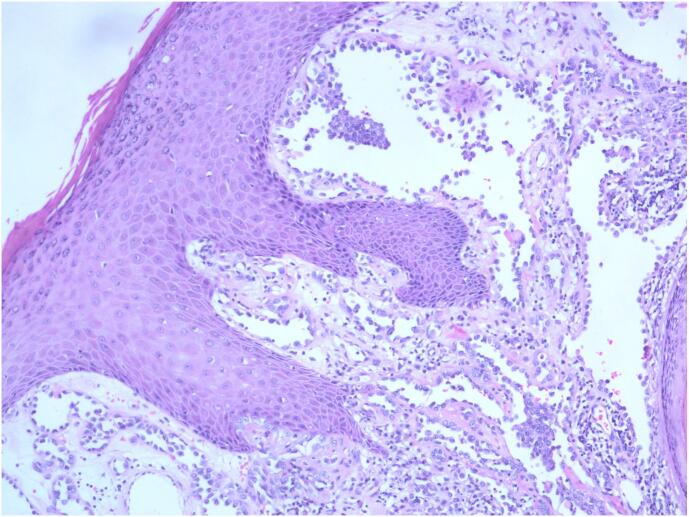
HE image, 200× magnification, the tumor is located in the subcutaneous dermis layer, consisting of arborizing blood vessels arranged in a testicular network pattern, lined with vascular endothelium resembling boot spikes; red blood cells are visible in the lumen, and local intraluminal papillary structures can be seen, similar to Dabska's tumor. (For interpretation of the references to colour in this figure legend, the reader is referred to the web version of this article.)

**Fig. 4:**
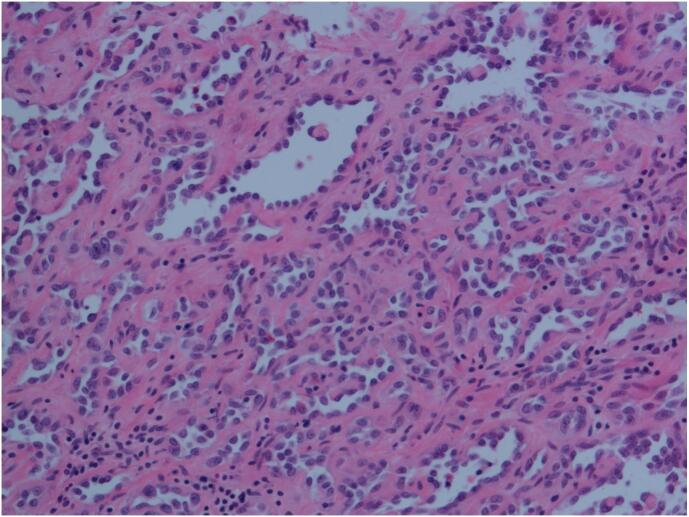
HE image, 200× magnification, The tumor shows a testicular network structure, with vascular endothelium arranged in a hobnailed pattern, collagen fibers were hyperplastic in the stroma, and lymphocytic infiltration was visible in the stroma.

**Fig. 5:**
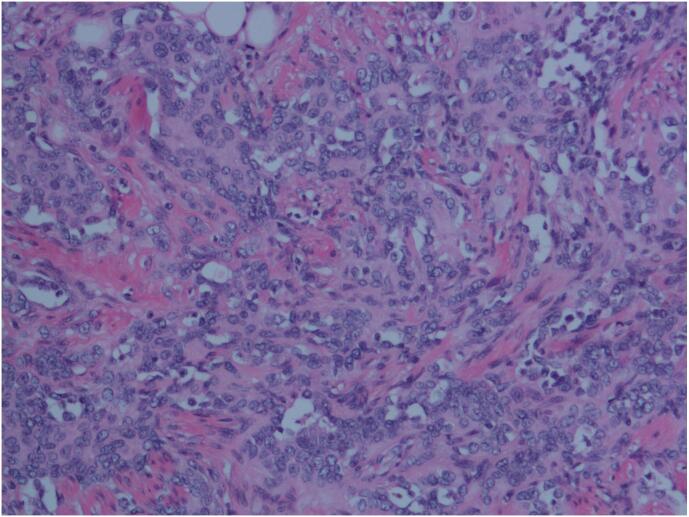
HE image, 200× magnification, the tumor grows in a solid nest-like, strip-like, and cord-like pattern, with whirlpool-like arrangement, vascular endothelial cell nuclei are round or oval, small nucleoli are visible, the cytoplasm is acidophilic and contains less cytoplasmic content, cell boundaries are unclear, and some cytoplasm appears vacuolated. Cytological atypia is minimal, mitotic figures are virtually absent, and the tumor infiltrates among collagen fibers in the dermis.

**Fig. 6:**
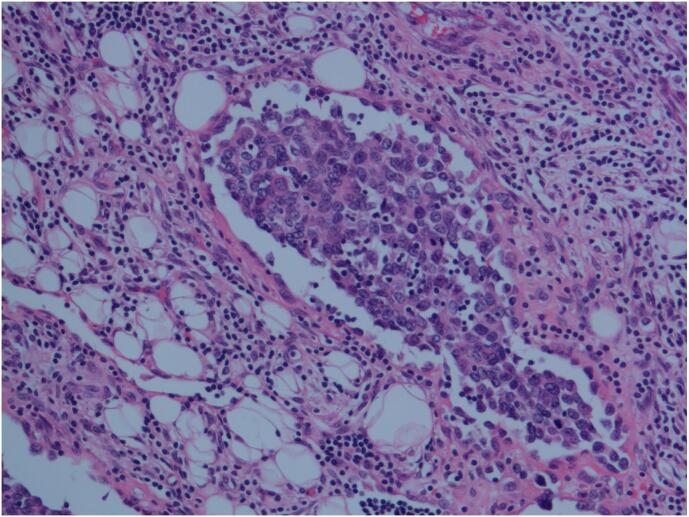
HE image, 200× magnification, intraluminal papillary structures, tumor cell nuclei are round, the stroma is accompanied by hyaline degeneration, similar to the morphology of Dabska's tumor, and a small number of lymphocytes are visible in the stroma.

Retiform hemangioendothelioma is characterized by distinctive branching, arborizing thin-walled vessels with endothelial cells arranged in a hobnailed pattern, similar to the structure of the testicular rete. Some areas may exhibit intraluminal papillary structures, and solid areas may show a cord-like or nest-like arrangement of epithelioid tumor cells. In some regions, dilated vascular lumens with intraluminal papillary clusters were observed, covered by hobnail-like endothelial cells with hyaline collagenous cores, which are morphologically similar to Dabska's tumor. The tumor cells infiltrate between collagen fibers in the dermis, with endothelial cells often arranged in a single layer in a columnar fashion, featuring round, deeply stained nuclei located at the top of the cells, and scant or indistinct cytoplasm, with rare mitotic figures. The tumor cells had lightly stained or eosinophilic cytoplasm, typically with less cytoplasmic content, a high nuclear-to-cytoplasmic ratio, and a transition between tumor cells and reticular vessels (Fig. 3, Fig. 4, Fig. 5, Fig. 6).

**Fig. 7:**
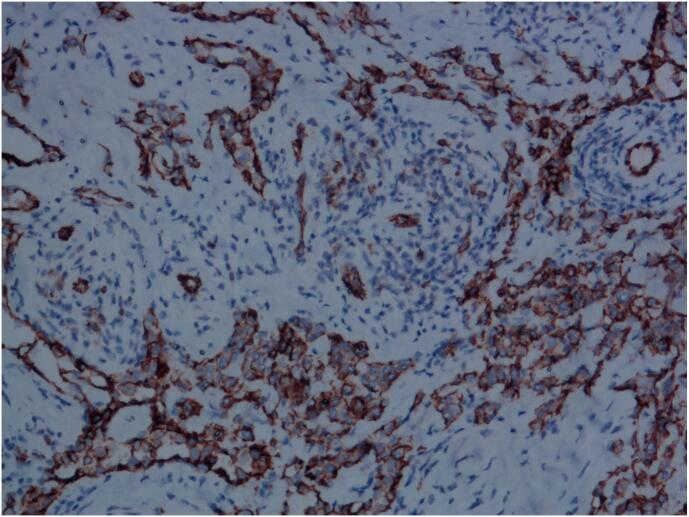
Immunohistochemistry image, CD31, 200× magnification, positive tumor vascular endothelial cells.

**Fig. 8:**
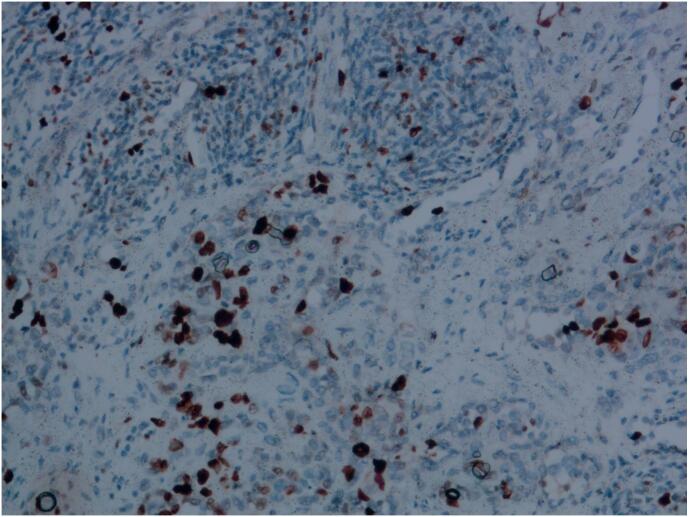
Immunohistochemistry image, CD34, 200× magnification, tumor cells were positive.

**Fig. 9:**
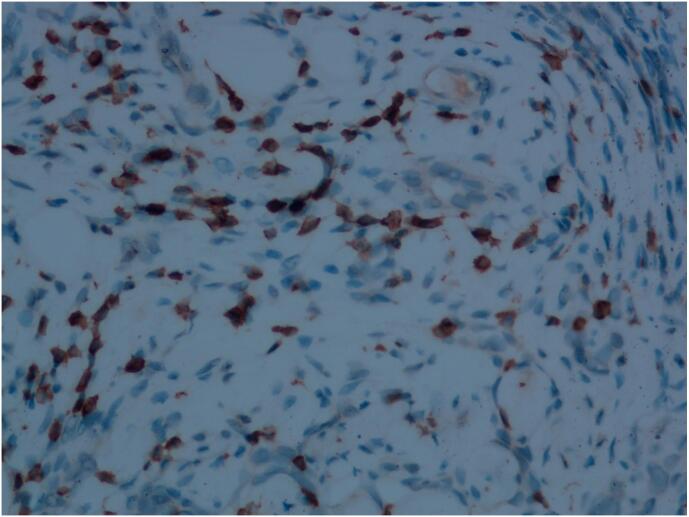
Immunohistochemistry image, 200× magnification, showing CD3 positive expression of T lymphocytes in the stroma.

Tumor cells expressed vascular endothelial markers such as CD31 (as shown in Fig. 7), CD34 (as shown in Fig. 8), ERG, and D2–40, with a high Ki67 proliferation index of approximately 40 %, and interstitial T lymphocytes expressed CD3 immunomarkers (as shown in Fig. 9). The work has been reported in line with the SCARE criteria [5].

After histological examination and immunohistochemistry, combined with a history of four recurrences, we made a definitive diagnosis of retiform hemangioendothelioma, with local malignancy transformed into angiosarcoma. The patient had enlarged lymph nodes in the neck area, suggesting a tumor lymph node metastasis. After extended resection, the patient received 14 sessions of radiotherapy over the following 3 months. The lymph nodes were significantly reduced in size, and a radiotherapeutic effect was evident. The patient is currently in good condition with no signs of recurrence.

## 3 Clinical discussion

Retiform hemangioendothelioma (RH) was first reported by Eduardo Calonje in 1994, who analyzed 15 cases of RH similar to angiosarcoma and considered RH to be low-grade angiosarcoma [1]. RH is an intermediate tumor with local invasiveness and rare metastasis, with an ICD-O code of 9136/1, and presents as a single, slowly enlarging mass or plaque-like tumor of the dermis and subcutaneous tissues. It is characterized by slender, elongated, arborizing, thin-walled vascular channels arranged in a retiform pattern. RH is very rare, with fewer than 50 cases reported domestically and internationally. The tumor is more common in young and middle-aged individuals, with an age range of 6–78 years, and is slightly more common in females. RH is more likely to occur in the distal extremities followed by the trunk, and a few cases can occur in the head and neck region. The case we report here is rare in the right submandibular region. Generally, RH has overlapping histological features with papillary intravascular endothelioma (Dabska's tumor), and Dabska and retiform hemangioendothelioma belong to the same tumor spectrum, both falling under the category of hobnail-like vascular endotheliomas. Typically, perivascular stroma is accompanied by CD3-positive T lymphocyte infiltration and may show collagenization.

The etiology of RH remains unclear. It shares some clinical features with Kaposi sarcoma, such as a slow-growing tumor with a high recurrence rate. Human herpesvirus (HHV-8) has been found in Kaposi's sarcoma associated with AIDS, and HHV-8 can also be present in non-AIDS-related Kaposi's sarcoma as well as in Castleman's disease. It has been reported that HHV-8 virus is also found in RH [2], and whether RH is related to HHV-8 virus requires further research. Another molecular event associated with RH is YAP1 gene fusion. In a study using targeted RNA sequencing, it was found that 5/13 (38 %) RH patients had rearrangements of the YAP1 gene, among which (2/13 cases) the fusion type of RH patients was YAP1-MAML2 fusion. In RH patients, YAP1 fusion tends to occur in males and in RH patients with lower limb involvement [6].

RH is a low-grade malignant tumor that is prone to recurrence, with 60 % of cases experiencing recurrence after many years. Individual cases of regional lymph node metastasis have been reported [7]. However, no cases of distant metastasis or death from the disease have been reported. In a report by Wang Jian on the clinical and pathological analysis of eight cases of retiform hemangioendothelioma [8], six cases were followed up for 1.5–5.5 years, with two cases of recurrence, one of which recurred twice within four and a half years. There have been no reported deaths associated with RH, the initial case series reported a median follow up of 7.35 years, with no mortality [[2], [3], [4]]. In our case, the RH patient experienced four recurrences over 20 years since the first excision and also experienced partial regional malignancy and enlargement of the cervical lymph nodes, indicating lymph node metastasis. Subsequently, the patient underwent radiotherapy (RT). Due to the high recurrence rate of RH, the best treatment plan is wide local excision, ensuring negative tumor margins. If regional lymph node metastasis is suspected, regional lymph node dissection can be considered, and adjuvant radiotherapy or chemotherapy can be used. There are also reports of using Mohs surgery for the treatment of RH [9].

Specifically, RH must be differentiated from the following types of tumors: 1. Dabska's tumor, which is more common in infants and children, usually occurs in the head and neck regions. Dabska's tumor has large dilated vessels and lacks slender branching testicular network vessels. Additionally, in terms of histology, Dabska's tumor has intravascular papillary structures, with tumor cells arranged in a single layer, exhibiting a boot-spike pattern. RH can also have this type of intravascular papillary structure, similar to Dabska's tumor; however, it is usually focal. 2. Angiosarcoma is a malignant tumor that generally occurs on the scalp of elderly individuals. Tumor vessels form irregular slit-like or sinusoidal structures, often anastomosing to form a traffic-like pattern, and exhibit infiltrative growth in the dermis. The tumor cells showed greater atypia than the retiform hemangioendothelioma, and mitotic figures were more easily seen. 3. Composite hemangioendothelioma, which often consists of RH as the main component, also includes other vascular tumor components, such as epithelioid hemangioendothelioma, spindle cell hemangioma, localized lymphangioma, angiomatosis, and angiosarcoma. In this case, the tumor component was mainly characterized by retiform testicular network structures and cord-like components, without the detection of other vascular tumor components.

## 4 Conclusion

RH is a low-grade malignant intermediate vascular tumor that is prone to recurrence and does not generally metastasize to distant sites. The histological features are characterized by unique branching, arborizing blood vessels arranged in a testicular network pattern, endothelial cells arranged in a hobnail pattern, with some areas showing dilated vascular lumens with intraluminal papillary clusters, morphologically similar to Dabska's tumor. Immunohistochemistry revealed CD31, CD34, ERG, D2–40, and other vascular immunohistochemical markers. The relationship between RH and HHV-8 virus is unclear, and some RH tumors have YAP1 gene rearrangements. Treatment is primarily based on wide excision. For cases with recurrent relapses that carry the risk of malignant transformation into angiosarcoma and some cases that develop regional lymph node metastasis after repeated recurrences, in addition to extended resection as a treatment option, postoperative radiotherapy and chemotherapy are also necessary.

## Author contribution

Lin Son made the data collection and data analysis, Dule Xing did the data interpretaton, Zhixin Cao and Yuanyuan Zong did the data collection and data anaysis, Dongsheng Hou design and write the paper.

## Consent

Written informed consent was obtained from the patient for publication of this case report and accompanying images. A copy of the written consent is available for review by the Editor-in-Chief of this journal upon request.

## Ethical approval

Ethical approval for this study was exempt from ethnical approval in our institution.

The institution name was: Medical Ethics Committee of Shandong Provincial Hospital Affiliated to Shandong First Medical University.

## Guarantor

Dongsheng Hou

## Sources of funding

This work was supported by Shandong Provinicial Medicine and Health Science and Technology Development Plan Project (grant numbers: 202001041369), and 10.13039/501100007129Shandong Provincial Natural Science Foundation (Grant numbers: ZR2020MH081).

## Declaration of competing interest

All authors have no financial and personal relationships with other people or organisations that could inappropriately influence their work.
